# Pronounced Fixation, Strong Population Differentiation and Complex Population History in the Canary Islands Blue Tit Subspecies Complex

**DOI:** 10.1371/journal.pone.0090186

**Published:** 2014-02-27

**Authors:** Bengt Hansson, Marcus Ljungqvist, Juan-Carlos Illera, Laura Kvist

**Affiliations:** 1 Department of Biology, Lund University, Lund, Sweden; 2 Island Ecology and Evolution Research Group, La Laguna, Tenerife, Spain; 3 Research Unit of Biodiversity, Oviedo University, Oviedo, Spain; 4 Department of Biology, University of Oulu, Oulu, Finland; BiK-F Biodiversity and Climate Research Center, Germany

## Abstract

Evolutionary molecular studies of island radiations may lead to insights in the role of vicariance, founder events, population size and drift in the processes of population differentiation. We evaluate the degree of population genetic differentiation and fixation of the Canary Islands blue tit subspecies complex using microsatellite markers and aim to get insights in the population history using coalescence based methods. The Canary Island populations were strongly genetically differentiated and had reduced diversity with pronounced fixation including many private alleles. In population structure models, the relationship between the central island populations (La Gomera, Tenerife and Gran Canaria) and El Hierro was difficult to disentangle whereas the two European populations showed consistent clustering, the two eastern islands (Fuerteventura and Lanzarote) and Morocco weak clustering, and La Palma a consistent unique lineage. Coalescence based models suggested that the European mainland forms an outgroup to the Afrocanarian population, a split between the western island group (La Palma and El Hierro) and the central island group, and recent splits between the three central islands, and between the two eastern islands and Morocco, respectively. It is clear that strong genetic drift and low level of concurrent gene flow among populations have shaped complex allelic patterns of fixation and skewed frequencies over the archipelago. However, understanding the population history remains challenging; in particular, the pattern of extreme divergence with low genetic diversity and yet unique genetic material in the Canary Island system requires an explanation. A potential scenario is population contractions of a historically large and genetically variable Afrocanarian population, with vicariance and drift following in the wake. The suggestion from sequence-based analyses of a Pleistocene extinction of a substantial part of North Africa and a Pleistocene/Holocene eastward re-colonisation of western North Africa from the Canaries remains possible.

## Introduction

**Figure 1:**
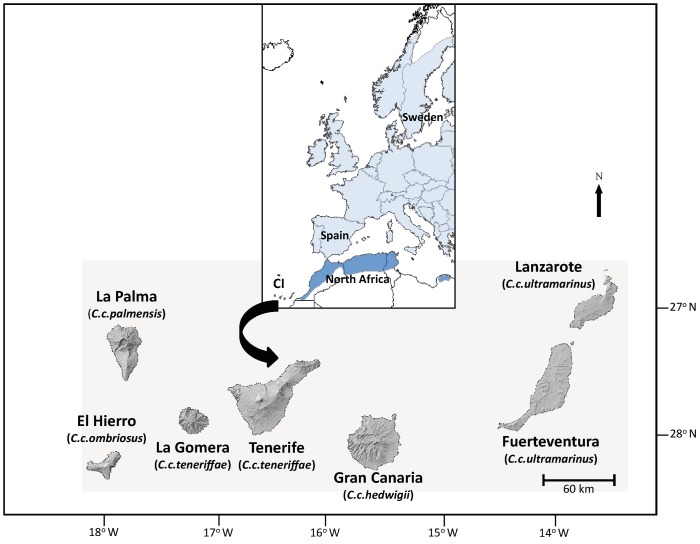
Map of the Canary Islands, Southern Europe, and northwestern Africa. Subspecies categorisation in blue tits according to Dietzen *et al*. (2008) is indicated.

Population divergence and speciation have fascinated evolutionary biologists ever since Darwin [1]. Divergence in allopatry, perhaps the most accepted mode of divergence, may occur over time in subdivided populations. Climatic cycles cause repeated range expansions and contractions of most populations, with vicariance, founder events as well as admixture following in the wake [2]. In birds, the genetic distances between newly diverged sister taxa translate to divergence times in the Pleistocene approximately 0.01–3.0 MYA [3], [4]. The ‘Pleistocene speciation hypothesis’ proposes that these speciation events occurred in isolated refugia over one to several full glacial cycles [5], [6]. A good model system to study genetic effects of isolation in the context of Pleistocene speciation model would be a system where several subpopulations or subspecies are available. Several such study systems are found on the Canary Islands [7], [8]. The Canary archipelago consists of seven large islands – from west to east: El Hierro, La Palma, La Gomera, Tenerife, Gran Canaria, Fuerteventura and Lanzarote – and are situated in the Atlantic, 100–500 km off the northwest coast of Africa (Figure 1). The islands are of increasing age from west to east (less than one up to twenty million years old) [9], [10]. The volcanic origin, the geographical situation (isolated but still relatively close to the mainland), the altitude (the highest peak is 3,718 m above sea level) and the absence of any land bridge connecting the archipelago with the continent, has led to a unique flora and fauna with a high degree of endemism [11], [12].

The blue tit (*Cyanistes caeruleus*) is found all over Europe, western Asia and northern Africa, including the Canary Islands [13]. Blue tits on the Canaries and North Africa are morphologically distinct from the European populations, and two subspecies complexes are distinguished: the European continental blue tits (the *caeruleus* group) and the Afrocanarian blue tit complex (the *teneriffae* group) [14]–[19]. Birds in the *teneriffae* group have dark blue or almost black cap, blue back and some populations lack the white wing pattern that is seen in the *caeruleus* group [20]. Further, the Afrocanarian complex have longer and thinner bills than the European subspecies (La Palma is an exception to this), shorter wings and longer tarsi [21] and their song differs to a great extent, even between the islands [22], [23]. Suggestions for giving *caeruleus* and *teneriffae* full species status have been put forward [14]–[19].

Studies of the blue tit radiation on the Canary Islands could potentially lead to new insights in the role of colonization, vicariance, populations size and drift in population divergence (cf. [24]). However, to be able to do so the historical colonization patterns and admixture events need to be understood. The closest mainland sources from where colonizing birds could come are north-western Africa (100 km; *C. c. ultramarinus*) and the southern Iberian peninsula (950 km; *C. c. ogliastrae*). Based on morphological data, Grant [21] proposed a historical demographic scenario following a classical stepping stone model, where the old eastern islands (Lanzarote and Fuerteventura) were colonized first from the African continent, then followed by the colonisation of the central islands (Gran Canaria, Tenerife, and La Gomera) and from there the western islands (El Hierro and La Palma). This scenario also included that the original populations on Lanzarote and Fuerteventura went extinct, and became recolonized from one of the central Islands, namely Tenerife [21]. Molecular studies of Canary Islands blue tits, analysing mitochondrial DNA (mtDNA) sequence data [15], [17]–[19] and recently also data from nuclear DNA (nDNA) [18], [19], interpret the results according to three different scenarios. The first interpretation follows Grant’s [21] original model and proposes that one of the central islands (Tenerife) was the first to be colonized from Africa, perhaps via Lanzarote and Fuerteventura (whose original populations went extinct), and from there all other islands were colonized, including Lanzarote and Fuerteventura [15], or alternatively these two populations were recolonized from Africa [17]. The second model suggests that the Canary Islands were colonized by blue tits from the African continent, but also from Europe [15], [17]. A main reason for this interpretation is that some island populations, in particular La Palma, share some specific genetic features with the European population (including a 12 bp mtDNA fragment) which are difficult to explain from the perspective of pure African colonisation [15]. However, recent work including additional North African populations does not support this model [18]. Finally, the third suggestion is that the Canaries represent an ancestral colonization from North Africa, and that these island populations later recolonized continental Africa after a large part of its original population had gone extinct [15], [18], [19]. This suggestion is particularly interesting since it would support a prominent role for small, peripheral populations as a source for wide-spread mainland populations, as opposed to the traditional view of such island populations forming population sinks (cf. [25], [26]). In line with this reasoning, other recent avian phylogenetic studies have also described systems where island populations may have acted as sources and contributed to the diversity of adjacent mainland populations [26]–[28]. Apart from these three main suggestions several other, not necessarily mutually exclusive, scenarios can be suggested, including that the Afrocanarian population was historically very large, holding high levels of genetic variation with at least occasional dispersal between islands and between islands and the mainland, and that relatively recent (Holocene) population declines and vicariance events have shaped the complex pattern of differentiation observed in sequence data of contemporary populations.

In the present study, we evaluate the degree of population genetic variation, fixation, differentiation and structure of the Canary Islands and North African blue tit subspecies complex using microsatellite markers and aim to get insights in the population history using coalescence based methods. In blue tits, an extensive number of nuclear microsatellites have been developed [29] and a first generation linkage map has been constructed [30]. This enabled us chose a set of markers with known linkage map position for our analyses. We analyse our genetic data using traditional methods based on predefined populations [31], as well as Bayesian clustering models [32], [33] and coalescence-based models [34], to explore the population history of Africanarian blue tits. These models allows testing different scenarios of population divergence, admixture and population size changes [34], and we were interested in evaluating the split between the European and the Afrocanarian lineages, the split between different Canary Islands, and, in particular, the split between the Canaries and North Africa.

## Materials and Methods

### Study Populations

This study included samples from the seven Canary Islands populations, as well as from North Africa (Morocco and Ceuta), and the mainland of Spain and Sweden (see **Figure 1**). In categorizing the island populations into subspecies, we followed Kvist *et al.* [15], Kvist [16] and Dietzen *et al.* [17]. According to Kvist [16] and Dietzen *et al.* [17] there are five subspecies breeding on the Canary Islands: *ombriosus* on El Hierro, *palmensis* on La Palma, *teneriffae* on La Gomera and Tenerife, *hedwigii* on Gran Canaria, and *ultramarinus* on Fuerteventura and Lanzarote. The subspecies *ultramarinus* is also found in North Africa, whereas *ogliastrae* occurs in Spain and *caeruleus* in Sweden. In Kvist *et al.* [15], the birds on Fuerteventura and Lanzarote were separated from North Africa and put into a separate subspecies, *degener*, but the newer data indicate that this may not be the case [16], [17]. The subspecies categorisation of Dietzen *et al.* [17] is shown on the map in **Figure 1**.

The birds were captured with mist-nets, measured and weighted, and a blood sample (≈30 µl) was taken by puncturing the brachial vein or with a syringe from the jugular vein. The blood samples were stored in a SET buffer containing 0.15 M NaCl, 0.05 M Tris and 0.001 M EDTA with a pH of 8.0, or in pure ethanol. The field work has been described elsewhere [15], [19]. The study and the protocols for handling and examining the birds were approved by the County Administrative Board and the Lund/Malmö Animal Review Board in Sweden, the Ministerio de Agricultura, Alimentacion y Medio Ambiente in Spain and the Haut Commissariat aux Eaux et Forets et a la Lutte Contre la Desertification in Morocco. After capture and examination the birds were immediately released into the wild.

### Molecular Markers and Genotyping

We selected a set of 21 microsatellite markers of which some were linked (located on the same chromosome) and some unlinked (situated on unique chromosomes) (**Table S1**) [38], [42], and genotyped a total of 206 blue tits. Summary statistics for all loci with their genomic location on the blue tit linkage map [30] and the zebra finch genome assembly [35] are given in **Table S1**. All birds were molecularly sexed by amplifying a Z- and W-linked locus, TGZ-002 (D. Dawson, University of Sheffield, unpublished), and this information was used for interpreting the genotypes of the Z-linked microsatellites.

All loci were PCR-amplified in three different multiplexes using QIAGEN Multiplex PCR kit (Qiagen, ltd.). Primer sequences and annealing temperatures for the microsatellites are given in Table S1 (see also Olano-Marin *et al.* [29] and Hansson *et al.* [30]). The PCR-products were separated and visualized using an ABI 3730 capillary sequencer (Applied Biosystems), and the genotypes were scored with Genemapper 4.0 (Applied Biosystems).

### Analyses of Population Fixation, Differentiation and Structure

To evaluate the genetic variability in each populations, we calculated number of alleles, allele richness, observed and expected heterozygosity [36] and F_IS_ [37] in Fstat ver. 2.9.3.2 [38]. We used this program also to evaluate deviations from Hardy–Weinberg equilibrium, calculated F_ST_ between all pairs of populations and tested these statistically (10,000 permutations; using a nominal level of 0.001).

In addition to F_ST_, Jost’s D_est_ was used as a measure of genetic differentiation between populations [31] and calculated for each population pair using the web based resource SMOGD v. 1.2.5 [39]. D_est_ is a relative measure of differentiation, which ranges from zero (no differentiation) to one (complete differentiation), and simulations have shown that it is an unbiased estimator of differentiation, and outperforms F_ST_, over a range of sample sizes and for markers with different numbers of alleles (including highly variable microsatellite loci) [40]. We used 1,000 bootstrap replicates and the harmonic mean of D_est_ across loci.

We evaluated population structure with the Bayesian clustering processes and MCMC simulations implemented in the program Structure [32]. By exploring a parameter space consisting of multi-locus allele frequencies of genetic clusters, the Structure algorithms search for clusters that maximize the likelihood that the observed individual genotypes belong to them. When there is strong population structure, the clusters that have the highest likelihood will coincide with a structure that minimizes deviations from Hardy–Weinberg equilibrium (single-locus measure) and linkage equilibrium (multi-locus measure) within the clusters. The most common and perhaps most biological meaningful way of running the Structure models is to use ‘admixture models’, which allow (but do not force) individuals to have a genetic origin from more than one genetic cluster [32]. For unlinked loci, any linkage disequilibrium in a data set is attributed to presently occurring substructuring. However, after an admixture event, linked loci will persist in linkage disequilibrium within a population for a time period that is inversely related to the rate of recombination between loci. When the genetic distance between loci is known, one can model linkage disequilibrium due to substructuring as well as due to linkage, and ‘linkage models’ that take both these types of linkage disequilibria into account have been implemented in later versions of Structure [33]. The linkage model can potentially provide additional information about the ancestry of individuals and may improve understanding complex relationships between populations [33]. We performed admixture and linkage models with Structure ver. 2.3.3 [32], [33]. The genetic distances between loci were provided (and came from the blue tit linkage map [30]) and allele frequencies were allowed to be correlated between populations in the models. For the full data set including 19 loci (i.e. excluding Tgu9 and Pca8; see below), we started each run with a burn-in period of 50,000 replicates, followed by a sampling period of 50,000 replicates. We also conducted separate analyses for the different linkage groups (LG1b, four loci; LG2, five loci; LGZ, five loci; **Table S1**) using a burn-in of 10,000 and a sampling period of 20,000 replicates. For each data set and model, we set the number of clusters (*K*) from 1 to 10, and used 20 iterations.

The most likely *K* was evaluated with the *ΔK*-method [41], where the change in log probability of data between two *K*s, and the variation in probability within a *K*, are used instead of the *K*-specific probability *per se* to evaluate which *K* has the strongest support from the data. *ΔK* was calculated with Structure Harvester (http://taylor0.biology.ucla.edu/struct_harvest/). The posterior probability of the admixture proportions of each individual from Structure was visualised using Distruct (N.A. Rosenberg, University of Michigan) and Clumpp [42].

Finally, we analyzed our data using the mtDNA subspecies classification as a prior. We performed these analyses following the subspecies structure suggested by Kvist *et al.* [15] and Dietzen *et al.* [17], respectively. We used admixture models with correlated allele frequencies, burn-in periods of 10,000, sampling periods of 20,000 replicates and 20 iterations. *K* was set to 7 (i.e. the number of subspecies). This procedure was used in order to evaluate whether the clustering process may be facilitated by such priors, and thus be able to support any of the suggested subspecies classifications.

### Analyses of Population History with Coalescence Models

We used the program DIYABC v. 1.0.4.45 [34] to explore the population history of blue tits. DIYABC is a coalescence-based program that infers the population history by looking backwards in time to examine genealogy of alleles until reaching the most recent common ancestor using approximate Bayesian computation algorithm. We chose 14 loci based on map distances at least 10 cM apart for diminishing any potential effect of linkage. The loci used were both autosomal (Cdi31-ZFM, CcaTgu19, CcaTgu21, Ase18, Pdoμ5, ApCo46-ZEST, LS2, PmaTGAn42, TG02-088, Titgata02) and sex-linked (Ase46-ZFM, CcaTgu31, TGZ-040, Phtr3). Based on previous studies [17], we started by building three different combinations of splitting between European, African and Canary Island populations, assuming no admixture after splitting events. We used the same mean mutation rate for all the loci, 10^−4^–10^−3^ [43], [44]. Priors for all three effective population sizes were 100–1,000,000 and for splitting times t_1_ and t_2_ in the past, 100–100,000 generations, depending on the population. We simulated 3,000,000 data sets, which were compared to the observed data to choose the scenario that best explains the data by estimating posterior probabilities for each scenario. The posterior probabilities were estimated using both, the logistic regression estimate and the direct estimate provided by the program. The logistic regression method was set to use from 30,000 to 60,000 first data sets (depending on the total number of simulations) as dependent variables and differences between observed and simulated data set summary statistics as the independent variables to perform a polychotomic weighted logistic regression. The intercept is then used as a point estimate. The direct estimate is based on the relative proportion of each scenario found in the 500 closest data sets. The scenario that explained the data the best was used to estimate divergence times and effective population sizes for North Africa and Europe.

Based on previous studies, we divided the Canary Island populations in three groups – western (La Palma, El Hierro), central (Tenerife, La Gomera, Gran Canaria) and eastern (Lanzarote, Fuerteventura) groups – in order to reduce the number of scenarios to be tested. We built separate scenarios including (i) western group with Europe and combined central group (**Figure S1**), (ii) central group and (iii) eastern group with Morocco (scenarios 2 and 3 in **Figure S2**). For eastern and central groups, we included also admixture into the scenarios. We simulated 4,000,000 data sets for scenario 1, 5,000,000 for scenario 2 and 6,000,000 for scenarios 3. Priors for effective population sizes were 100–10,000 individuals and for splitting times 100–1,000,000 generations, again depending on the population. The best scenarios from these runs (see below) included simultaneous splits within the eastern, central and western islands and the European population as an outgroup. These were included in the final run, where we built six scenarios differing in relation to when the splits occurred and from where the branch leading to eastern islands originate. All islands were treated as distinct populations and 6,000,000 simulations were run.

## Results

### Genetic Variation, Fixation and Differentiation

Two of the microsatellite loci included in the present study did not amplify in at least one population: Tgu09 did not amplify in samples from the Canary Islands and North Africa, and Pca8 did not amplify in the samples from La Palma (**Table S1**). The reason for this is most likely that there are substantial primer site mutations leading to amplification failure in these populations. These two loci were excluded from further analyses, but we note that the pattern observed for Tgu09 supports a relationship between populations on the Canaries and North Africa.

For the remaining 19 loci, the gene diversity, allele richness, and number of alleles were highest for the three mainland populations, Sweden, Spain and North Africa (**Table 1**). Observed and expected heterozygosity were similar, F_IS_ was low in all cases, and there were no statistical deviations from Hardy–Weinberg equilibrium within populations (**Table 1**).

**Table 1:** Genetic characteristics of blue tit populations at 19 microsatellite loci including number of genotyped individuals, number of alleles, number of monomorphic loci, number of unique alleles, allelic richness, observed heterozygosity (H_O_), expected heterozygosity (H_E_) and F_IS_.

Population	Sample size	Mean numberof alleles	Number of monomorphic loci	Number ofunique alleles	Allelerichness	H_O_	H_E_	F_IS_
Sweden	20	7.5	1	22	5	0.65	0.63	−0.03
Spain	22	7.3	0	16	5	0.61	0.61	−0.01
El Hierro	13	2.5	9	3	2	0.26	0.28	0.02
La Palma	24	3.4	6	16	3	0.31	0.33	0.05
La Gomera	21	3.5	7	4	3	0.34	0.34	−0.01
Tenerife	25	5.1	3	6	4	0.45	0.46	0.01
Gran Canaria	22	4.8	4	7	3	0.48	0.46	−0.05
Fuerteventura	20	3.8	6	6	3	0.31	0.36	0.13
Lanzarote	17	1.9	7	3	2	0.27	0.25	−0.05
North Africa	22	5.8	1	11	4	0.58	0.54	−0.05

There was strong fixation and differentiation between most populations with F_ST_-values ranging between 0.022 for Sweden and Spain and 0.636 for El Hierro and La Palma, and with D_est_-values ranging between 0.015 for Sweden and Spain and 0.793 for La Palma and Tenerife (**Table 2**). The pair-wise F_ST_- and D_est_-values were highly correlated (Pearson’s correlation: *r* = 0.667). All F_ST_-values were significantly different from zero (*p*<0.001).

**Table 2:** F_ST_-values (below the diagonal) and D_est_ *-*values (above diagonal) between blue tit populations based on genotypic data from 19 loci.

	Swe	Spa	El Hie	La Pal	La Gom	Ten	Gr Can	Fue	Lan	N Afr
Sweden	*	0.015	0.604	0.667	0.684	0.504	0.504	0.625	0.600	0.425
Spain	0.022	*	0.557	0.711	0.633	0.473	0.518	0.642	0.581	0.459
El Hierro	0.421	0.415	*	0.725	0.232	0.194	0.354	0.300	0.324	0.305
La Palma	0.454	0.469	0.636	*	0.712	0.793	0.737	0.622	0.619	0.567
La Gomera	0.445	0.435	0.453	0.614	*	0.250	0.440	0.387	0.374	0.337
Tenerife	0.325	0.321	0.325	0.571	0.341	*	0.205	0.371	0.456	0.184
Gran Canaria	0.322	0.333	0.428	0.559	0.439	0.252	*	0.547	0.574	0.479
Fuerteventura	0.406	0.410	0.444	0.577	0.481	0.389	0.447	*	0.167	0.180
Lanzarote	0.442	0.431	0.552	0.635	0.538	0.467	0.515	0.374	*	0.277
North Africa	0.245	0.263	0.350	0.469	0.366	0.234	0.331	0.243	0.319	*

### Population Structure using Structure

**Figure 2:**
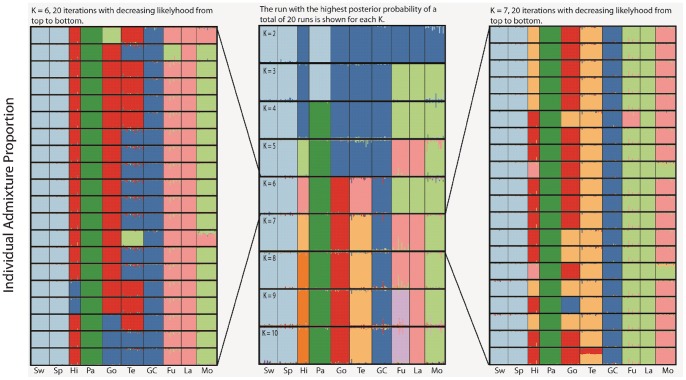
Admixture proportions, i.e. proportion of membership to each of *K* clusters (indicated with different colours), of individual blue tits from different populations. Results are from admixture models in Structure using the full set of 19 loci. The central graph shows the results for each *K*, from *K* = 2 to 10 (the run with the highest posterior probability of a total of 20 runs are shown for each *K*). All 20 iterations for *K* = 6 is shown to the left, and for *K* = 7 to the right (sorted according to declining posterior probability from top to bottom). Populations are, from left to right: Sweden (Sw), Spain (Sp), El Hierro (Hi), La Palma (Pa), La Gomera (Go), Tenerife (Te), Gran Canaria (GC), Fuerteventura (Fu), Lanzarote (La) and Morocco (Mo).

Structure analyses supported the presence of strong population differentiation (**Figure 2**). Admixture models showed very high membership assignment of specific individuals and populations independently of which *K* is modelled, but weak consistency in clustering of some populations within and between *K*s. This has probably to do with the fact that there is a high degree of fixation of unique alleles in the populations on the Canary Islands (**Table 1**), and that some populations are sharing skewed allele frequencies with a few other populations with different combinations of populations for different loci. The relationship between the central island populations (La Gomera, Tenerife and Gran Canaria) and El Hierro was difficult to disentangle with different combinations of populations clustering in different iterations. Moreover, the two eastern islands (Fuerteventura and Lanzarote) and Morocco showed weak clustering with partly consistent clustering (in particular al low *K*s), whereas the two European populations showed highly consistent clustering, and La Palma a consistent unique lineage in the majority of iterations (**Figure 2**).

**Figure 3:**
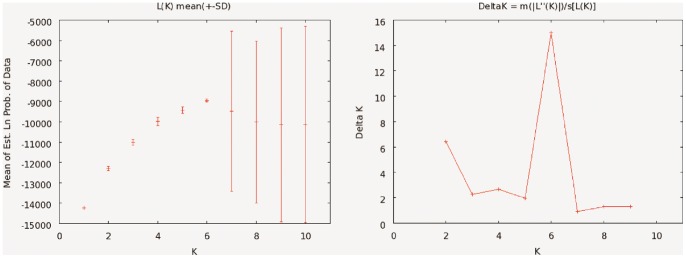
Summary of the clustering result from the population structure analysis using the program structure 2.3.3. (a) Mean likelihood (± SD) for different number of clusters, *K*. (b) *ΔK*-values for different *K*s; suggesting *K = *6 as the most likely structure.

The *ΔK*-method suggested 6 clusters as the most likely population structure (**Figure 3**), but the low *ΔK* for *K* = 7 was mainly an effect of a single run with very low posterior probability (Ln(P) = −26,148); the other 19 runs had a higher mean posterior probability (Ln(P) = −8,601) than the 20 iterations at *K* = 6 (Ln(P) = −8,950). However, also for the most likely *K* there was striking variation in how some of the populations clustered between different runs (compare the 20 iterations for *K* = 6 in **Figure 2**), despite the fact that these different runs had very similar likelihoods (**Figure 3**).

The Structure analyses using linkage models, where the genetic distances between linked loci were taken into account, gave very similar results compared to the admixture analyses. This was true for the full data set of 19 loci as well as for the three linkage groups separately. Thus, modelling linkage did not provide any additional resolution of the genetic relationship between populations (data not shown).

We also performed admixture models using the subspecies classifications suggested by Kvist *et al*. [25] and Dietzen *et al*. [27] as priors. *K* was set to 7 (i.e. the number of suggested subspecies) and 20 iterations were performed. These analyses were made because we were interested in evaluating whether detecting a structure corresponding to the subspecies categorisations would be facilitated by using such priors. However, the substructure groupings suggested by Kvist *et al.* [15] and by Dietzen *et al.* [17] were only partly supported: most strikingly *teneriffae* on La Gomera and *teneriffae* on Tenerife did not cluster together more often than in runs without priors, *teneriffae* (*hedwigii*) on Gran Canaria did not cluster with *teneriffae* on Tenerife, and *ultramarinus*/*degener* in North Africa, Lanzarote and Fuerteventura did not cluster in these runs (**Figure S3**).

### Population History using DIYABC

**Figure 4:**
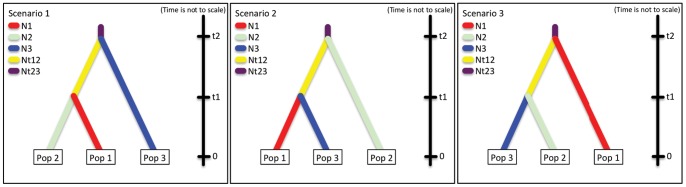
Graphical illustration of three historical events tested with DIYABC. Pop 1 = Europe (Spain and Sweden), Pop2 = All Canary Islands, and Pop 3 = North Africa (Morocco).

The first scenario (Europe, Canaries and Africa) that we tested in DIYABC indicated that the European *caeruleus* population is an outgroup for the Canary Island and African populations. Both the logistic approach and the direct approach supported the scenario 3 in **Figure 4** (posterior probabilities 1.000 and 0.666, respectively). The mode for divergence between European and Canary Island+African blue tits was 19,000 generations ago (95% highest posterior density, HPD, 6,922–93,900) and between Canary Islands and Morocco 2,840 generations ago (95% HPD 1,010–53,400).

In the runs of western, central and eastern island groups, respectively, the logistic regression method supported simultaneous splits of the island populations from their common ancestors within these groups (scenario 4 in **Figure S1**; scenario 5 in **Figure S2**), with no admixture after the divergence (posterior probabilities 0.963–1.000). The direct approach, on the other hand, did not give strong support for any of the scenarios (posterior probabilities very even for all scenarios). Divergence times for a split between Fuerteventura, Lanzarote and Morocco were estimated to be only 120 generations ago (95% HPD 107–1,610 generations), between La Gomera, Tenerife and Gran Canaria 100 generations ago (95% HPD 100–270 generations) and between La Palma, El Hierro and Central Islands 1,710 generations ago (95% HPD 465–44,100 generations).

**Figure 5:**
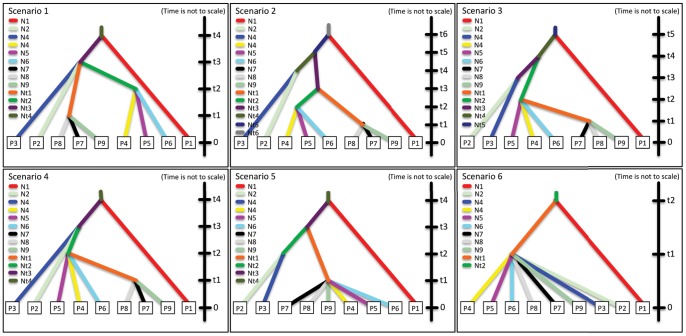
Graphical illustration of six historical events tested with DIYABC. Pop 1 = Europe (Spain and Sweden), Pop 2 = El Hierro, Pop 3 = La Palma, Pop 4 = La Gomera, Pop 5 = Tenerife, Pop 6 = Gran Canaria, Pop 7 = Lanzarote, Pop 8 = Fuerteventura, and Pop 9 = North Africa (Morocco).

The final run included these simultaneous divergence events within island groups, but with different branching orders. Best support with the direct approach was given to a model where all Canary Island and North African populations diverged simultaneously (scenario 6 in **Figure 5**, posterior probability 0.296). Logistic regression method gave the best support (posterior probability 0.261, scenario 2 in **Figure 5**) for the scenario including the most recent split between the eastern Islands and Morocco and a previous split between the central islands Tenerife, La Gomera and Gran Canaria from a common branch that had separated from a branch leading to La Palma and El Hierro, i.e. the eastern group and central group share a common ancestor. Divergence of Central Islands occurred after divergence of western islands La Palma and El Hierro. However, the support for these scenarios was only marginally stronger than for the other scenarios (posterior probabilities for the different scenarios varied between 0.091 and 0.296).

## Discussion

Our results complement the findings from previous mtDNA and nDNA studies of the Canary Islands blue tit radiation [15], [17]–[19] by showing that there is low genetic variation and strong fixation within island populations and high differentiation between populations at a larger set of nuclear microsatellite loci. The western and central Canary Islands populations, El Hierro, La Palma, La Gomera, Tenerife and Gran Canaria, stand out as very different genetically, and assessing how they are related is problematic. The eastern islands, Lanzarote and Fuerteventura, cluster with North Africa in admixture models with lower number of genetic clusters. These results only partly reflect the results from studies applying other loci [15], [17]–[19]. That different sets of markers and loci are showing partly different patterns of population relationships is in line with expectations from drift scenarios with incomplete lineage sorting [45], [46].

In an aim to achieve increased resolution of potential admixture events, we applied linkage models in Structure. The linkage model accounts for allelic correlations between linked loci that arise both due to population structure and due to admixture, i.e. ‘admixture linkage disequilibrium’ [41]. Admixture linkage disequilibrium will persist in a population for some time after an admixture event due to the reduced recombination between linked loci. Applying such models when using linked markers could improve the clustering process [33]. However, this was not the case in our analyses; the results from the admixture and the linkage models were similar. Perhaps our markers were not linked sufficiently close (map distance between adjacent markers ranged between 2 and 50 cM; Table 1), implying that any admixture linkage disequilibrium would have been broken down quite rapidly after an admixture event, or perhaps any admixture events took place a long time ago. Similar outcomes for admixture and linkage models could also arise if the differentiation that we observed today are mainly caused by drift and fixation in isolation e.g. due to vicariance, rather than by colonisation and admixture.

The Canary Islands were formed 1 to 20 MYA [20], [21], and the phylogeny reconstructions by Illera et al. [19] and Päckert et al. [18] suggest that the blue tit colonisation dates back 3 to 4 MYA. Our reconstructions of the population history in DIYABC suggest that the European population is an outgroup to the Afrocanarian population, and that the populations within the western, central and eastern groups split simultaneously. This result differs to some extent from those of the phylogeographical reconstructions, which show ancient splits between some islands – in particular, between La Palma and the other islands [18], [19]. Our models suggest that most of the splits were comparably recent; assuming a generation time of c. 2 year, our divergence time estimates ranged from c. 38,000 (14,000–188,000) years between the European and Afrocanarian clades to c. 240 (200–3,200) years for the different central islands and for the eastern islands and North Africa. These timing estimates are based on rates of 10^−4^–10^−3^ mutations per generation, i.e. commonly employed microsatellite mutation rates [43], [44]. However, our timing estimates are much more recent than those being estimated with phylogenetic reconstructions [18], [19], which indicates that the microsatellite loci we have screened have lower mutation rates than commonly assumed.

The pronounced divergence with low genetic diversity and yet unique genetic material in the Canary Island system requires an explanation. The population at La Palma is particularly intriguing since it shows highly divergent patterns on our sets of markers as well as on mtDNA and nDNA sequence data [15], [17]–[19]. La Palma (i) shares a unique 12 bp mtDNA (control region) fragment with the European population which is not present in the other Afrocanarian populations (which indicates a Eurasian ancestry at this locus), (ii) differs with 34 nucleotides to the Gran Canaria population at the cytochrome *b* (1,005 bp) [17] and at 26 nucleotides to Tenerife at the control region (539 bp) [15], (iii) has six loci with fixed alleles and 16 unique alleles at the 19 loci we have studied (and at locus Pca8 it is the only population with null alleles), and (iv) shares null-alleles at the Z-linked marker Tgu09 with all other Afrocanarian populations (which indicates Afrocanarian ancestry). Interestingly, a recent phylogenetic study that included a previously unstudied Libyan population of blue tits (subspecies *cyrenaicae* in the *teneriffae* group) in the eastern part of the species’ Afrocanarian range, clustered the Libyan population as a basal lineage of *C. teneriffae* together with La Palma [18]. These two eastern and western peripheral populations at the Afrocanarian range shared all major indels at the studied sequences and differed in this respect from the other taxa in the Afrocanarian group. Such large genetic distances over a widespread area suggests an old history of isolation. The remote location of La Palma northwest of the other islands has implied only sporadic inflow of immigrants. Also the Libyan population shows distinct allopatry, being located east of the nearest African population in Tunisia (see Figure 1; [18]). It is worth noticing that La Palma has low but not the lowest degree of microsatellite genetic variation, which may suggest a relatively large long-term population size.

In conclusion, most mtDNA and nDNA haplotypes [15], [18], [19] and several microsatellite alleles [19] [this study] are unique to the Canaries and are not found on the mainland populations. A phylogeographical scenario that could explain the substantial amount of genetic variation that is observed today among all the different populations in the *teneriffae* complex is the presence of a historically much larger Afrocanarian population in which genetic variation was generated and maintained (cf. [47]). Such a population could have been distributed over a substantial part of North Africa and the Canaries. Indeed, North Africa has been going through several cycles of climatic shifts, including a major transition from wet to dry habitat after the last glaciation [48], with possible strong impact on the amount of suitable habitat for a forest species like the blue tit. Historical dispersal events within and between the Afrocanarian region and a relatively recent population contraction in North Africa resulting in vicariance, drift, lineage sorting and extinctions and recolonizations could have resulted in the complex population structures that we observe today. In line with recent promising undertakings by Illera et al. [19] and Päckert et al. [18], further phylogenetic and coalescent reconstructions using sequence data from a larger set of nuclear loci and populations will be decisive to understand the processes that shaped the blue tit diversity in the region. Such information could confirm the interesting possibility that some of the Canary Islands populations have acted as a source for the current populations on the western parts of North African. This would be of particular interest since it would highlight the importance of small, island populations as sources for mainland colonisations, as opposed to the traditional view of such peripheral populations acting as population sinks (cf. [18], [19], [25], [26]).

## Supporting Information

Figure S1 **Graphical illustration of five historical events tested with DIYABC.** Pop 1 = Europe (Spain and Sweden), Pop 2 = La Palma, Pop 3 = El Hierro, Pop 4 = Central Islands (La Gomera, Tenerife, Gran Canaria).
(TIFF)

Figure S2 **Graphical illustration of six historical events tested with DIYABC.** Test of the eastern Islands and North Africa: Pop 1 = Fuerteventura, Pop 2 = Lanzarote, and Pop 3 = North Africa (Morocco). Test of the Central Islands: Pop 1 = Tenerife, Pop 2 = Gran Canaria, and Pop 3 = La Gomera.
(TIFF)

Figure S3 **Proportion of membership of individual blue tits of different populations to** ***K*** **= 7 clusters corresponding to the subspecies categorisation of (a) Kvist** ***et al*** **. (2005) and (b) Dietzen** ***et al*** **. (2008).** All 20 iterations for *K* = 7 are shown (sorted according to declining posterior probability from top to bottom). Results are from admixture models using the full set of 19 loci. Populations are, from left to right: Sweden (Sw), Spain (Sp), El Hierro (Hi), La Palma (Pa), La Gomera (Go), Tenerife (Te), Gran Canaria (GC), Fuerteventura (Fu), Lanzarote (La) and Morocco (Mo).
(EPS)

Table S1 **Genomic location of 21 microsatellite loci on the blue tit linkage map and the zebra finch genome assembly.** Total number of alleles, and average observed heterozygosity (H_O_), expected heterozygosity (H_E_) and F_IS_, in the populations are given, followed by primer sequences and annealing temperatures (Ta; TD indicates touch-down PCR).
(PDF)

## References

[1] Darwin C (1859) On the Origin of Species by Means of Natural Selection. London: John Murray.

[2] Hewitt GM (1996) Some genetic consequences of ice ages, and their role in divergence and speciation. Biological Journal of the Linnean Society 58: 247–276.

[3] Johnson NK, Cicero C (2004) New mitochondrial DNA data affirm the importance of Pleistocene speciation in North American birds. Evolution 58: 1122–1130. 15212392 10.1111/j.0014-3820.2004.tb00445.x

[4] Weir JT, Schluter D (2007) The latitudinal gradient in recent speciation and extinction rates of birds and mammals. Science 315: 1574–1576. 17363673 10.1126/science.1135590

[5] Mila B, McCormack JE, Castaneda G, Wayne RK, Smith TB (2007) Recent postglacial range expansion drives the rapid diversification of a songbird lineage in the genus *Junco* . Proceedings of the Royal Society of London B 274: 2653–2660. 10.1098/rspb.2007.0852 PMC2279216 17725978

[6] Mila B, Smith TB, Wayne RK (2007) Speciation and rapid phenotypic differentiation in the yellow-rumped warbler *Dendroica coronata* complex. Molecular Ecology 16: 159–173. 17181728 10.1111/j.1365-294X.2006.03119.x

[7] Illera JC, Emerson BC, Richardson DS (2007) Population history of Berthelot’s pipit: colonisation, gene flow and morphological divergence in Macaronesia. Molecular Ecology 16: 4599–4612. 17908210 10.1111/j.1365-294X.2007.03543.x

[8] Suárez NM, Betancor E, Klasert TE, Almeida T, Hernándes M, et al (2009) Phylogeography and genetic structure of the Canarian common chaffinch (*Fringilla coelebs*) inferred with mtDNA and microsatellite loci. Molecular Phylogenetics and Evolution 53: 556–564. 19632343 10.1016/j.ympev.2009.07.018

[9] Anguita F, Hernan F (1986) Geochronology of Some Canarian Dike Swarms - Contribution to the Volcano-Tectonic Evolution of the Archipelago - Discussion. Journal of Volcanology and Geothermal Research 30: 155–158.

[10] Coello J, Cantagrel JM, Hern†n F, F£ster JM, Ibarrola B, et al (1992) Evolution of the eastern volcanic ridge of the Canary Islands based on new K-Ar data. Journal of Volcanology and Geothermal Research 53: 251–274.

[11] Juan C, Emerson BC, Oromi P, Hewitt GM (2000) Colonization and diversification: towards a phylogeographic synthesis for the Canary Islands. Trends in Ecology and Evolution 15: 104–109. 10675925 10.1016/s0169-5347(99)01776-0

[12] Illera JC, Rando JC, Richardson DS, Emerson BC (2012) Age, origins and extinctions of the avifauna of Macaronesia: a synthesis of phylogenetic and fossil information. Quaternary Science Reviews 50: 14–22.

[13] del Hoyo J, Elliott A, Christie DA (2007) Handbook of the Birds of the World (HBW), Band 12: Picathartes to Tits and Chickadees. Barcelona: Lynx Edicions.

[14] Salzburger W, Martens J, Sturmbauer C (2002) Paraphyly of the Blue Tit (*Parus caeruleus*) suggested from cytochrome b sequences. Molecular Phylogenetics and Evolution 24: 19–25. 12128025 10.1016/s1055-7903(02)00265-8

[15] Kvist L, Broggi J, Illera JC, Koivula K (2005) Colonisation and diversification of the blue tits (*Parus caeruleus teneriffae*-group) in the Canary Islands. Molecular Phylogenetics and Evolution 34: 501–511. 15683925 10.1016/j.ympev.2004.11.017

[16] Kvist L (2006) Response to “Taxonomic status of ‘phylogroups’ in the *Parus teneriffae* complex (Aves)” by George Sangster. Molecular Phylogenetics and Evolution 38: 290. 10.1016/j.ympev.2005.10.009 16314112

[17] Dietzen C, Garcia-del-Rey E, Castro GD, Wink M (2008) Phylogeography of the blue tit (*Parus teneriffae*-group) on the Canary Islands based on mitochondrial DNA sequence data and morphometrics. Journal of Ornithology 149: 1–12.

[18] Packert M, Martens J, Hering J, Kvist L, Illera JC (2013) Return flight to the Canary Islands–the key role of peripheral populations of Afrocanarian blue tits (Aves: *Cyanistes teneriffae*) in multi-gene reconstructions of colonization pathways. Molecular Phylogenetics and Evolution 67: 458–467. 23454090 10.1016/j.ympev.2013.02.010

[19] Illera JC, Koivula K, Broggi J, Packert M, Martens J, et al (2011) A multi-gene approach reveals a complex evolutionary history in the *Cyanistes* species group. Molecular Ecology 20: 4123–4139. 21880092 10.1111/j.1365-294X.2011.05259.x

[20] Harrap S, Quinn D (1996) Tits, nuthatches & treecreepers London: Christopher Helm, cop.

[21] Grant PR (1979) Ecological and Morphological Variation of Canary Island Blue Tits, *Parus caeruleus* (Aves, Paridae). Biological Journal of the Linnean Society 11: 103–129.

[22] Schottler B (1993) Canary Islands blue tits (*Parus caerulesus* ssp.): Differences and variation in territorial song: Preliminary results. Boletim do Museu Municipal do Funchal Suppl. 2: 273–277.

[23] Schottler B (1995) Songs of blue tits *Parus caeruleus palmensis* from La Palma (Canary Islands): A test of hypotheses. Bioacoustics 6: 135–152.

[24] Price T (2008) Speciation in Birds. Greenwood Village, Colorado: Roberts and Company.

[25] Bellemain E, Bermingham E, Ricklefs RE (2008) The dynamic evolutionary history of the bananaquit (*Coereba flaveola*) in the Caribbean revealed by a multigene analysis. BMC Evolutionary Biology 8: 240. 18718030 10.1186/1471-2148-8-240 PMC2533019

[26] Sheldon FH, Lohman DJ, Lim HC, Zou F, Goodman SM, et al (2009) Phylogeography of the magpie-robin species complex (Aves: Turdidae: Copsychus) reveals a Philippine species, an interesting isolating barrier and unusual dispersal patterns in the Indian Ocean and Southeast Asia. Journal of Biogeography 36: 1070–1083.

[27] Filardi CE, Moyle RG (2005) Single origin of a pan-Pacific bird group and upstream colonization of Australasia. Nature 438: 216–219. 16281034 10.1038/nature04057

[28] Jonsson KA, Fabre PH, Ricklefs RE, Fjeldsa J (2011) Major global radiation of corvoid birds originated in the proto-Papuan archipelago. Proceedings of the National Academy of Science USA 108: 2328–2333. 10.1073/pnas.1018956108 PMC3038755 21262814

[29] Olano-Marin J, Dawson D, Girg A, Hansson B, Ljungqvist M, et al (2009) A genome-wide set of 104 microsatellite markers for the blue tit (*Cyanistes caeruleus*). Molecular Ecology Resources 10: 516–532. 21565051 10.1111/j.1755-0998.2009.02777.x

[30] Hansson B, Ljungqvist M, Dawson DA, Mueller JC, Olano-Marin J, et al (2010) Avian genome evolution: insights from a linkage map of the blue tit (*Cyanistes caeruleus*). Heredity 104: 67–78. 19707235 10.1038/hdy.2009.107

[31] Jost L (2008) GST and its relatives do not measure differentiation. Molecular Ecology 17: 4015–4026. 19238703 10.1111/j.1365-294x.2008.03887.x

[32] Pritchard JK, Stephens M, Donnelly P (2000) Inference of population structure using multilocus genotype data. Genetics 155: 945–959. 10835412 10.1093/genetics/155.2.945 PMC1461096

[33] Falush D, Stephens M, Pritchard JK (2003) Inference of population structure using multilocus genotype data: linked loci and correlated allele frequencies. Genetics 164: 1567–1587. 12930761 10.1093/genetics/164.4.1567 PMC1462648

[34] Cornuet J-M, Ravigne V, Estoup A (2010) Inference on population history and model checking using DNA sequence and microsatellite data with the software DIYABC (v1.0). BMC Bioinformatics 11: 401. 20667077 10.1186/1471-2105-11-401 PMC2919520

[35] Warren WC, Clayton DF, Ellegren H, Arnold AP, Hillier LW, et al (2010) The genome of a songbird. Nature 464: 757–762. 20360741 10.1038/nature08819 PMC3187626

[36] Nei M (1987) Molecular Evolutionary Genetics. New York: Columbia Univ. Press.

[37] Weir BS, Cockerham CC (1984) Estimating F-statistics for the analysis of population structure. Evolution 38: 1358–1370. 28563791 10.1111/j.1558-5646.1984.tb05657.x

[38] Goudet J (2002) FSTAT, A Program to Estimate and Test Gene Diversities and Fixation Indices, Version 2.9.3.2. Available from http://www2.unil.ch/popgen/softwares/fstat.htm.

[39] Crawford NG (2010) SMOGD: software for the measurement of genetic diversity. Molecular Ecology Resources 10: 556–557. 21565057 10.1111/j.1755-0998.2009.02801.x

[40] Gerlach G, Jueterbock A, Kraemer P, Deppermann J, Harmand P (2010) Calculations of population differentiation based on GST and D: forget GST but not all of statistics! Molecular Ecology. 19: 3845–3852. 10.1111/j.1365-294X.2010.04784.x 20735737

[41] Evanno G, Regnaut S, Goudet J (2005) Detecting the number of clusters of individuals using the software STRUCTURE: a simulation study. Molecular Ecology 14: 2611–2620. 15969739 10.1111/j.1365-294X.2005.02553.x

[42] Jakobsson M, Rosenberg NA (2007) CLUMPP: a cluster matching and permutation program for dealing with label switching and multimodality in analysis of population structure. Bioinformatics 23: 1801–1806. 17485429 10.1093/bioinformatics/btm233

[43] Ellegren H (2004) Microsatellites: simple sequences with complex evolution. Nature Reviews Genetics 5: 435–445. 10.1038/nrg1348 15153996

[44] Sun JX, Helgason A, Masson G, Ebenesersdottir SS, Li H, et al (2012) A direct characterization of human mutation based on microsatellites. Nature Genetics 44: 1161–1165. 22922873 10.1038/ng.2398 PMC3459271

[45] Clegg S (2010) Evolutionary changes following island colonization in birds. In: Losos JB, Ricklefs RE, editors. The theory of island biogeography revisited. Princeton, New Jersey: Princeton University Press. 293–325.

[46] Chaves JA, Parker PG, Smith TB (2012) Origin and population history of a recent colonizer, the yellow warbler in Galápagos and Cocos Islands. Journal of Evolutionary Biology 25: 509–521. 22239606 10.1111/j.1420-9101.2011.02447.x

[47] Hartl DL, Clark AG (1997) Principles of population genetics. Sunderland, Massachusetts: Sinauer Associates, Inc.

[48] Kropelin S, Verschuren D, Lezine AM, Eggermont H, Cocquyt C, et al (2008) Climate-driven ecosystem succession in the Sahara: the past 6000 years. Science 320: 765–768. 18467583 10.1126/science.1154913

