# Supplementary figures and images for: Pronounced Fixation, Strong Population Differentiation and Complex Population History in the Canary Islands Blue Tit Subspecies Complex
Source: PLoS One. 2014 Feb 27;9(2):e90186. doi: 10.1371/journal.pone.0090186 (PMC3937385; doi:10.1371/journal.pone.0090186)

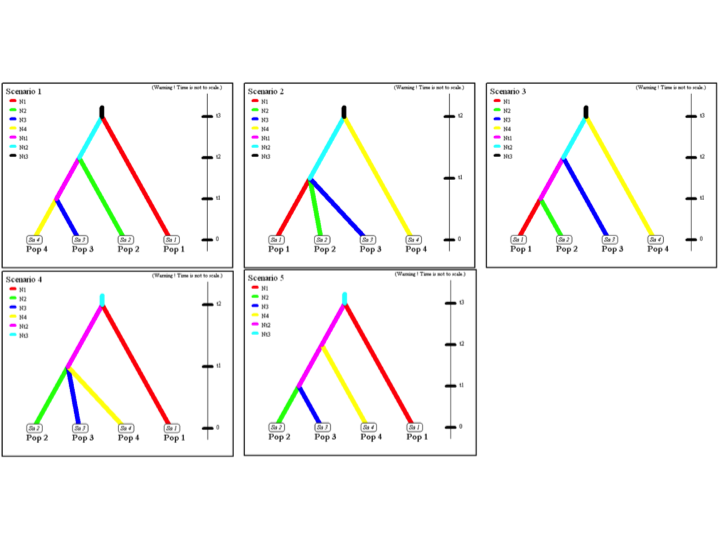

Supplement: Figure S1 — Graphical illustration of five historical events tested with DIYABC. Pop 1 = Europe (Spain and Sweden), Pop 2 = La Palma, Pop 3 = El Hierro, Pop 4 = Central Islands (La Gomera, Tenerife, Gran Canaria). (TIFF) [file pone.0090186.s001.tiff]

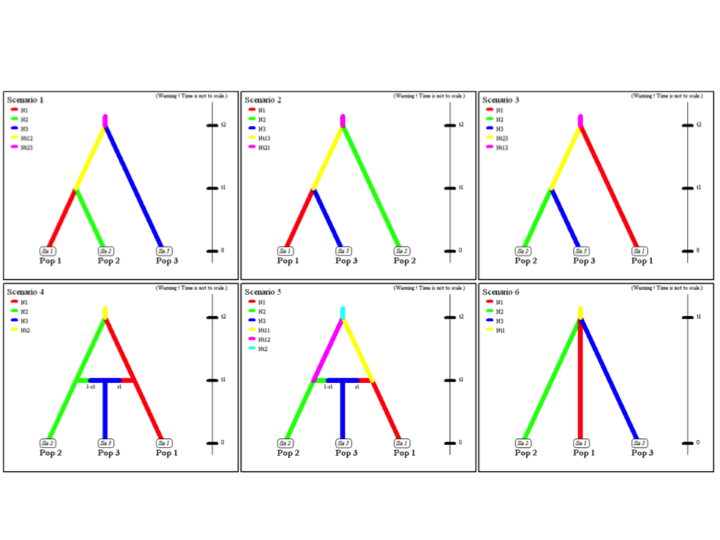

Supplement: Figure S2 — Graphical illustration of six historical events tested with DIYABC. Test of the eastern Islands and North Africa: Pop 1 = Fuerteventura, Pop 2 = Lanzarote, and Pop 3 = North Africa (Morocco). Test of the Central Islands: Pop 1 = Tenerife, Pop 2 = Gran Canaria, and Pop 3 = La Gomera. (TIFF) [file pone.0090186.s002.tiff]
