# Supplementary material for: Pronounced Fixation, Strong Population Differentiation and Complex Population History in the Canary Islands Blue Tit Subspecies Complex
Source: PLoS One. 2014 Feb 27;9(2):e90186. doi: 10.1371/journal.pone.0090186 (PMC3937385; doi:10.1371/journal.pone.0090186)
Supplement: Table S1 — Genomic location of 21 microsatellite loci on the blue tit linkage map and the zebra finch genome assembly. Total number of alleles, and average observed heterozygosity (HO), expected heterozygosity (HE) and FIS, in the populations are given, followed by primer sequences and annealing temperatures (Ta; TD indicates touch-down PCR). (PDF) [file pone.0090186.s004.pdf]

Table S1. Genomic location of 21 microsatellite loci on the blue tit linkage map and the zebra finch genome assembly. Total number of alleles, and average observed heterozygosity (H_O), expected heterozygosity (H_E) and $F_{IS,}$ in the populations are given, followed by primer sequences and annealing temperatures (Ta; TD indicates touch-down PCR).

Locus	Accession number	Blue tit linkage group	Linkage map position	Zebra finch chromosome	Chromosome position	No. of alleles	H_O	H_E	F_{IS}	5' - 3' F-Primer seq	Ta
Cdi31-ZFM	AB089172.1	7	78	7	4,201,012	27	0.57	0.58	0.02	F: GAACTTCTGCATTTGTTCCTCTC	56
										R: GAGAGCGTGCTGAATGAGTG	
CcaTgu19	DV579042.1	10	0	10	6,370,604	30	0.76	0.78	0.03	F: CTGGACCATGACTGCAAGATT	TD 61-53
										R: CAGTGGCAAAKCAGCACCT	
CcaTgu21	DV961016.1	11	27	11	12,285,425	7	0.10	0.09	-0.09	F: GGCAGACATGATTGCATCC	TD 61-53
										R: TCTCAGTGGTCATTGGAAAGTG	
Ase18	AJ276375.1	3a	0	3	13,906,080	12	0.38	0.35	-0.08	F: ATCCAGTCTTCGCAAAAGCC	60
										R: TGCCCCAGAGGGAAGAAG	
Pdoμ5	Y15126.1	4b	0	4	48,504,861	29	0.59	0.61	0.04	F: GATGTTGCAGTGACCTCTCTTG	54
										R: GCTGTGTTAATGCTATGAAAATGG	
ApCo46-ZEST	AF520885.1	1b	109	1	104,151,755	4	0.28	0.26	-0.08	F: GCTGCCAGCACTCTGAATGTC	57
										R: GATTCAGCAAAATAGGGGTCAGAAG	
LEI160	AM159172.1	1b	132	1	109,699,352	4	0.18	0.15	-0.18	F: GCAGACAGCCGTTAATATATGCG	60
										R: AACCAAAACACAAGCTCTTGCA	
PmaD22	AY260527	1b	139	1	112,574,840	45	0.73	0.73	0.01	F: GATCAGAGCTTGCCTCAACAC	57
										R: TCTGGGCTGAAATACCTACCC	
LS2	NA	1b	141	NA	NA	7	0.42	0.42	-0.01	F: CTCAAACTGAAAATGTAGATTCACC	TD 60-50
										R: TTTACATTTTTTCCCATGAGGC	
PmaTGAn42	AY260540.1	2	115	2	70,321,386	18	0.73	0.74	0.02	F: ACTTCCACATGCCAGTTTCC	57
										R: TGTTAAGGCAGAGAGGTGGG	
POCC6	U59117.1	2	125	2	44,644,858	25	0.68	0.68	0.01	F: TCACCCTCAAAAACACACACA	TD 60-53
										R: ACTTCTCTCTGAAAAGGGGAGC	
Pca8	AJ279810	2	134	2	38,606,246	49	0.80	0.80	0.00	F: ACTTCTGAAACAAAGATGAAATCA	57
										R: TGCCATCAGTGTCAAACCTG	
TG02-088	DV579347.1	2	151	2	93,538,047	7	0.33	0.33	0.02	F: TGTGTGTTGACAGTATTCTCTTGC	55
										R: TTTAAACCTAATAAACGTCACACAGTC	
PmaGAn11	AY260531.1	2	177	2	107,642,995	4	0.13	0.18	0.26	F: GCTTCTGCCTCCATTAAGAGTC	57
										R: GAAAAATCACCCACTCAGCC	

Titgata02	AY792958.1	2	181	2	108,368,370	35	0.83	0.83	-0.01	F: TCTTTTGGGTTGCCTGAAGT	TD 60-50
										R: TTGTTGCAACTACAAGACATTTGA	
Ase46-ZFM	AJ276775.1	Z	0	Z	32,967,367	13	0.41	0.46	0.10	F: CTGGCTGTATCTTGGTGTGC	56
										R: GCTAACTTTCCATTGAACTGTCC	
CcaTgu31	CK311793.1	Z	50	Z	44,295,294	8	0.32	0.30	-0.08	F: AGCCCAGATTKGAAATRAACT	55
										R: TGTAGCYYTGTAGAATTAGCAAA	
TGZ-037	DV945670.1	Z	74	Z	54,801,985	4	0.04	0.03	-0.06	F: AAAACACCTTGTAATTTAAAACTGG	55
										R: CATAGATACATATCAATACAGCACATTC	
TGZ-040	DV949035.1	Z	80	Z	52,307,759	9	0.45	0.39	-0.14	F: AAAAGTCTTTCTGGACTGTGCT	55
										R: AAAATACAACAGACATAGGCATACA	
Phtr3	AM056070.1	Z	129	Z	13,746,684	9	0.16	0.16	0.03	F: ATTTGCATCCAGTCTTCAGTAATT	TD 60-50
										R: CTCAAAGA AGTGCATAG AGATTTCAT	
Tgu09	CK307658.1	Z	142	Z	70,750,908	5	0.09	0.09	-0.01	F: GGGGACGTTTTATCTGTTACC	56
										R: GCAGTGCCCAAGTTCAGAGT	
